# Impact of congenital syphilis incidence on congenital heart surgery outcomes in Brazil

**DOI:** 10.21542/gcsp.2025.17

**Published:** 2025-05-15

**Authors:** Gabriel Kaleb Martins, Maria Rayane Félix Pacífico

**Affiliations:** Institute of Medical Education, Bahia, Brazil

## Abstract

This study examines the impact of congenital syphilis incidence trends on congenital heart surgery outcomes in Brazil, with a focus on mortality rates, treatment costs, and length of hospital stay. Using an ecological study design, we analyzed nationwide data from 2007 to 2023 to identify regional and temporal patterns in these relationships. The results revealed significant increases in the incidence of syphilis across all regions, accompanied by marked disparities in healthcare resource allocation and surgical capacity. The Northeast and Southeast regions demonstrated expansion in approved surgeries, while the North and South regions exhibited comparatively slower growth, highlighting inequities in specialized care access. Correlation analyses identified a moderate negative association between syphilis incidence and mortality rate, and a strong negative correlation between cost per surgery and mortality. Our regression model explained 44.2% of the variance in mortality rates, with syphilis incidence, cost per surgery, and length of hospital stay emerging as significant predictors. Machine learning validation confirmed the predominance of linear relationships, with Linear Regression (*R*^2^ = 0.513) outperforming Random Forest (*R*^2^ = 0.321). These findings highlight the need for targeted interventions to address regional disparities and optimize resource allocation, offering evidence-based guidance to policymakers and healthcare providers to improve neonatal health outcomes in Brazil.

## Introduction

Cardiovascular surgery, particularly in the realm of congenital heart defects (CHDs), is one of the most intricate and high-stakes disciplines in modern medicine. CHDs, structural abnormalities present at birth, are among the most common types of birth defects, and are a leading cause of neonatal morbidity and mortality worldwide^1^. These defects encompass a wide spectrum, ranging from relatively simple conditions, such as atrial septal defects, to complex malformations, such as hypoplastic left heart syndrome, each necessitating tailored surgical interventions^2^. The management of CHDs demands not only technical precision but also a comprehensive understanding of the interplay between prenatal factors, perioperative care, and long-term outcomes^3^. Infectious diseases during pregnancy, such as congenital syphilis (CS), have emerged as critical contributors to the complexity and severity of congenital heart defects^4^. Despite being preventable through early detection and treatment, the resurgence of CS in recent years has raised significant concerns regarding its implications for cardiovascular surgery, particularly in low- and middle-income countries where healthcare resources may be limited^5^.

The intersection of CS and cardiovascular surgery presents unique challenges for both clinicians and healthcare systems. Infants born with CS often exhibit a spectrum of complications, including hepatosplenomegaly, anemia, and neurological impairments, which can complicate preoperative assessments and increase operative risks^6^. Moreover, CS has been associated with specific cardiac anomalies, such as aortic insufficiency, coronary ostial stenosis, and structural defects that necessitate surgical correction^7^. For cardiovascular surgeons, these cases require meticulous planning and multidisciplinary collaboration to address coexisting conditions and optimize surgical outcomes^8^. However, the increasing incidence of CS places additional strain on healthcare systems, potentially affecting resource allocation, surgical capacity, and the quality of care delivered^9^. This underscores the need for a focused investigation into how trends in CS incidence influence surgical outcomes, including mortality rates, treatment costs, and postoperative recovery dynamics.

This study aims to bridge a critical gap in the literature by examining the relationship between CS incidence and congenital heart surgery outcomes, with a particular emphasis on variables directly relevant to cardiology and cardiovascular surgery. The primary objective is to determine whether rising syphilis incidence correlates with worsened surgical outcomes and to explore potential regional and temporal variations in these relationships. By leveraging population-level data from publicly available health databases in Brazil from 2007 to 2023, this study seeks to provide actionable insights for improving neonatal health outcomes. The ecological study design allows for the exploration of macro-level associations, offering valuable insights into how regional disparities in syphilis incidence may influence surgical outcomes across different healthcare contexts^10^. Although ecological studies cannot establish causality, they are instrumental in generating hypotheses and identifying patterns that can guide further research or public health interventions^11^.

From a cardiology perspective, this research holds significant implications for understanding the broader impacts of CS on neonatal cardiovascular health. For instance, the identification of syphilis-related risk factors for adverse surgical outcomes can inform prenatal screening protocols and early intervention strategies, reducing the burden of complex surgeries later in life^12^. Similarly, insights into cost drivers and recovery challenges can help optimize resource allocation and improve the efficiency of healthcare delivery, ensuring that cardiovascular surgery programs are better equipped to meet the needs of affected populations^13^. Furthermore, the study’s findings contribute to the global discourse on CS and neonatal health, offering lessons that can be applied to other regions facing similar challenges^14^. Ultimately, this research underscores the importance of addressing CS as a public health priority, not only to reduce the direct burden of the disease but also to mitigate its broader impacts on neonatal cardiovascular health and healthcare systems^15^.

By focusing specifically on the interplay between CS and cardiovascular surgery outcomes, this study provides a foundation for evidence-based strategies to enhance surgical success rates, reduce mortality, and improve long-term quality of life for infants born with congenital heart defects. The integration of advanced analytical techniques, including ordinary least squares regression and machine learning models, ensures a robust and nuanced understanding of these dynamics, enabling the identification of key predictors and potential areas for intervention^16^. As cardiovascular surgery continues to evolve, addressing the systemic and epidemiological factors that influence surgical outcomes will be essential for achieving equitable and sustainable improvements in neonatal health^17^.

## Methodology

This study was designed to investigate the relationship between CS incidence and congenital heart surgery outcomes, with a focus on mortality rates, treatment costs, and length of hospital stay. The research follows an ecological study design, as it examines population-level data aggregated by region and year, rather than individual-level data. This approach allows for the exploration of trends and associations at a macro level, providing insights into how regional variations in syphilis incidence may influence surgical outcomes across Brazil from 2007 to 2023. Although ecological studies cannot establish causality, they are valuable for generating hypotheses and identifying patterns that can inform further research or public health interventions.

The study utilized routinely collected health data from three primary sources, all publicly available and covering the entire population of Brazil. The first dataset was extracted from the Sistema de Informação de Agravos de Notificação (SINAN), which provides annual counts of confirmed CS cases by region. This dataset served as the foundation for calculating the incidence of syphilis, expressed as cases per 1,000 live births. The second dataset was obtained from the Sistema de Informações sobre Nascidos Vivos (SINASC), which records the annual number of live births by region. This information was critical for normalizing syphilis cases and ensuring that incidence rates were comparable across regions and years. The third dataset was obtained from the Sistema de Informações Hospitalares (SIH-SUS), which includes detailed information on congenital heart surgeries, such as the number of surgeries performed, average cost per surgery, length of stay (LOS), and mortality rates. These datasets collectively provided a comprehensive overview of the variables of interest, syphilis incidence, healthcare resource utilization, and surgical outcomes, at the population level.

To ensure the quality and usability of the data, extensive pre-processing was performed. Missing or inconsistent entries were identified and removed to minimize errors in subsequent analyses. For example, any region-year combination with >20% missing entries in syphilis cases, live births, or surgical metrics was excluded to avoid bias, and as such data previous of the national implantation of the more rigorous data system in 2007 were excluded. Variables were also transformed to ensure compatibility across datasets. Monetary values were adjusted to 2023 Brazilian Reais using the National Consumer Price Index (IPCA) from Brazil’s Institute of Geography and Statistics (IBGE). Costs from earlier years were inflated using the formula: Adjusted Cost=Original cost*(2023 cost/IPCA). Feature engineering was another essential step in this process. New variables were created to facilitate analysis, including the syphilis incidence rate, calculated as (Syphilis_Cases / Live_Births) * 1000. Similarly, cost per surgery was derived by dividing total costs by the number of surgeries performed in each region and year, while average LOS was calculated as the total LOS divided by the number of surgeries. Once individual datasets were preprocessed, they were merged based on two key identifiers: Region and Year. This ensured that all variables were aligned temporally and geographically, enabling region-specific and year-specific analyses. The resulting unified dataset provided a robust foundation for subsequent exploratory and statistical analyses.

Exploratory data analysis was conducted to gain insights into trends and patterns in the data before proceeding to formal modeling. Time-series plots were generated to visualize changes in syphilis incidence, mortality rates, treatment costs, and LOS over the study period. These visualizations helped to identify regional and temporal variations, such as increasing syphilis incidence rates in certain regions and declining mortality rates over time. Descriptive statistics, including measures of central tendency and dispersion, were calculated for each variable to understand their distributions and identify potential outliers. Correlation analysis was also performed to examine pairwise relationships between variables using Pearson’s correlation coefficient. This step aimed to uncover significant associations that could inform the selection of variables for subsequent modeling. By conducting an EDA, the study ensured that the data were thoroughly understood and prepared for more advanced analyses.

To quantify the relationship between syphilis incidence and surgical outcomes, two complementary statistical modeling approaches were employed. The first approach utilizes ordinary least squares (OLS) regression, a traditional statistical method well suited for identifying linear relationships. In this model, the dependent variable was mortality rate, whereas the independent variables included syphilis incidence rate, cost per surgery, and LOS. OLS regression was chosen for its interpretability and ability to provide clear estimates of the strength and direction of the relationships between variables. The second approach employed Random Forest regression, a machine-learning technique capable of capturing nonlinear relationships and interactions between variables. This model was used to predict mortality rates based on the same predictors as in the OLS model. Random Forest regression was selected for its robustness and ability to handle complex data structures, providing a complementary perspective to the OLS results, and the linear model was described in terms of its power. Both models were rigorously evaluated using standard metrics, including mean squared error (MSE) and *R*^2^ score, to assess their performance and explanatory power.

To optimize the performance of the Random Forest model in predicting congenital heart surgery mortality rates, a grid search with five-fold cross-validation was conducted to tune key hyperparameters. The search space included the number of trees in the forest (n_estimators: 100, 200, 500), maximum depth of the trees (max_depth: 5, 10, 20, none), minimum number of samples required to split an internal node (min_samples_split: 2, 5, 10), and the minimum number of samples required to be at a leaf node (min_samples_leaf: 1, 2, 4). These parameters were selected to control the model complexity, reduce overfitting, and capture potential nonlinear interactions among the predictors.

The best-performing model was obtained using the following configuration: n_estimators = 200, max_depth = 10, min_samples_split = 5, and min_samples_leaf = 2. This setup achieved a mean squared error of 4.64 and an *R*^2^ score of 0.321, representing the best trade-off between model bias and variance. The coefficient of determination (*R*^2^) was used as the evaluation metric, as the outcome variable was continuous (mortality rate).

Ethical considerations were addressed throughout the study to ensure compliance with ethical guidelines. All data were anonymized and publicly available, eliminating the need for individual consent. The study adhered to the RECORD (Reporting of Studies Conducted Using Observational Routinely Collected Health Data) checklist, which emphasizes transparency in reporting observational research using routinely collected data. Potential biases, such as underreporting of syphilis cases or regional disparities in data quality, were acknowledged and discussed in the limitations section. By addressing these ethical and methodological considerations, the study ensured that its findings are both reliable and reproducible.

This ecological study design is particularly suited for exploring population-level trends and generating hypotheses regarding the relationship between CS incidence and congenital heart surgery outcomes. While it does not allow for causal inferences, it provides a valuable foundation for understanding broader patterns and informing future research and public health interventions.

## Results

**Figure 1.**
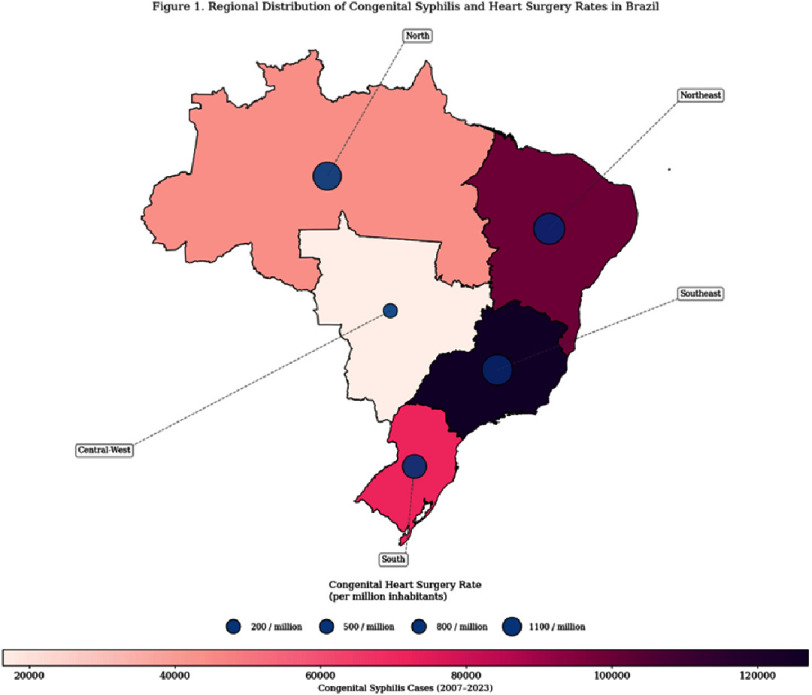
This graphic demonstrates the rising regional trends in congenital syphilis cases from 2007 to 2023 and the rate of congenital heart surgery in each region. The Southeast and Northeast regions consistently report the highest burdens (darker colors), while surgical capacity (bubbles) remains disproportionately low in high-risk areas, such as the North and Central-West.

The results of this study are presented through a detailed exploration of temporal trends, regional disparities, and statistical relationships between CS incidence and congenital heart surgery outcomes. Figure 1 reveals pronounced regional disparities in congenital syphilis cases across Brazil from 2007 to 2023, with the Southeast and Northeast regions reporting the highest cumulative incidence. Overlaying proportional bubbles further highlights stark inequities in congenital heart surgery rates, where the Southeast region dominates the largest bubble, contrasting sharply with the Central-West and North regions. This divergence suggests a misalignment between disease burden and healthcare capacity, particularly in northern and central-western areas, which face higher syphilis rates but have limited access to specialized surgical care (Figure 1).

**Figure 2.**
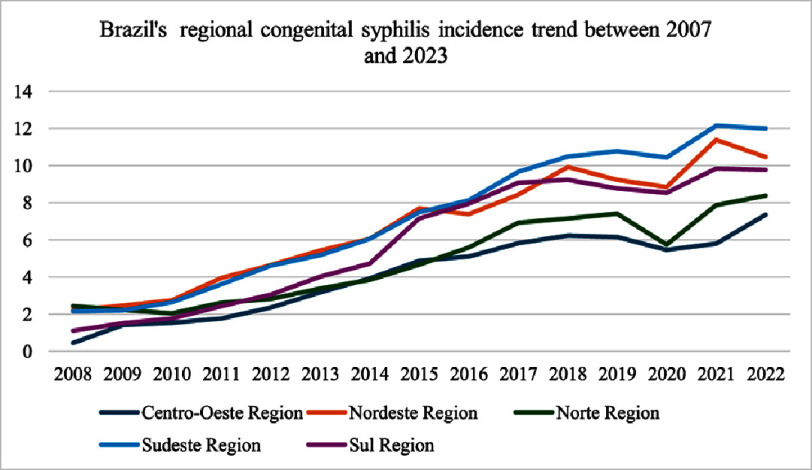
The rising trend of all regions for congenital syphilis. Despite a temporary decrease in 2020 due to COVID-19, the trend has continued.

Figure 2 shows an alarming escalation in syphilis incidence rates across all regions from 2007 to 2023, with significant regional variations. For instance, the Central-West Region has experienced a nearly 16-fold increase in syphilis incidence, rising from 0.449 cases per 1,000 live births in 2008 to 7.349 cases in 2022. This rapid surge highlights a growing public health challenge in the region, potentially driven by systemic barriers such as limited healthcare infrastructure or socioeconomic disparities. Similarly, the Northeast Region exhibited a dramatic rise, increasing from 2.218 cases in 2008 to 10.458 cases in 2022. The North Region also saw substantial growth, rising from 2.436 cases in 2008 to 8.378 cases in 2022. In contrast, the Southeast and South Regions demonstrated more moderate but still significant increases, with rates increasing to 12.006 and 9.759 cases per 1,000 live births, respectively. These trends suggest that regions with higher population density or better healthcare infrastructure, such as the Southeast, may face different challenges compared to less densely populated areas, such as the North or Central-West. The sharp increase in the incidence of syphilis underscores the urgent need for targeted interventions to address this growing burden.

**Figure 3.**
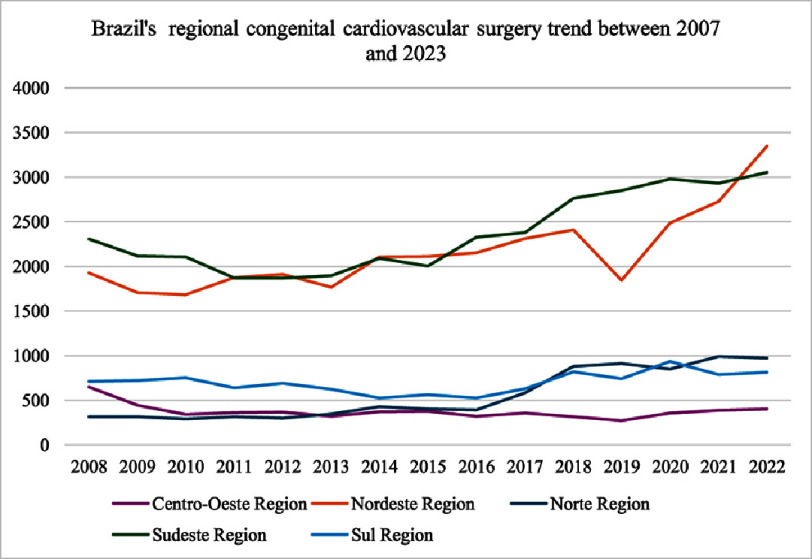
Absolute values of congenital cardiovascular surgery per geographic surgery. The principal interpretation is that the Northeast and Southeast regions have a tendency to offer that procedure, while the other regions rise but at a lower rate and yet at relatively low amounts.

Complementing these findings, Figure 3 shows regional variations in the number of surgery-approved congenital heart surgeries over time, reflecting differences in healthcare resource allocation and surgical capacity. The Northeast Region has consistently reported the highest number of approved surgeries, ranging from 1,727 in 2008 to 3,347 in 2022, underscoring its role as a hub for congenital heart surgery in Brazil. The Southeast Region followed closely, with numbers increasing from 2,161 in 2008 to 3,052 in 2022, reflecting its robust healthcare infrastructure and specialized medical facilities. In contrast, the North and South Regions exhibited more modest growth, with the North reporting an increase from 425 surgeries in 2008 to 973 in 2022, as shown in Table 1, and the South rising from 738 to 814 surgeries over the same period. The Central-West Region, despite its smaller population, decreased the number of surgeries from 701 in 2008 to 403 in 2022. These trends suggest that regions with higher syphilis incidence rates, such as Northeast and Central-West, may have received additional funding or support to expand their surgical capacity, enabling them to address the growing demand for congenital heart surgeries. However, the slower growth in North and South raises concerns about potential inequities in resource allocation or systemic barriers limiting access to specialized care in these regions.

**Table 1:** Summary of key descriptive statistics for the main variables analyzed in this study. For each variable, measures such as mean, standard deviation, quartiles, and median were provided to describe their distributions across the dataset. These statistics demonstrate the central tendencies, variability, and ranges of the data, facilitating a better understanding of populationlevel trends.

**Variable**	**Mean**	**Standard deviation**	**1** ^**st**^ ** quartile**	**2** ^**nd**^ ** quartile**	**Median**	**3** ^**rd**^ ** quartile**	**4** ^**th**^ ** quartile**
**Syphilis** ** Cases**	3431.20	3276.36	6.00	1087.00	2241.00	5129.00	11891.00
**Live** ** Births**	1,070,126.00	2,430,437.00	220,168.00	308,375.00	888,268.00	1,152,846.00	17,733,934.00
**Surgeries**	2306.78	5515.83	272.00	425.00	849.00	2111.00	37,697.00
**Cost per surgery**	2985.26	927.00	1264.20	2297.29	2941.42	3534.44	5279.30
**Length of stay**	11.32	1.77	8.10	10.10	11.10	12.30	17.70
**Mortality Rate**	7.95	1.87	3.87	6.72	7.71	9.04	14.24
**Syphilis** ** Incidence Rate**	5.13	3.38	0.03	2.23	4.86	7.87	12.14

In addition to the trends identified in Table 1, a broader regional perspective is crucial to better contextualize the disparities in cardiovascular surgical care delivery across Brazil. While Table 1 highlights the correlations between mortality and hospitalization expenditures, a more detailed breakdown of key indicators by region can provide a comprehensive understanding of structural inequalities in the healthcare system. Table 2 presents a comparative summary of five relevant indicators from 2008 to 2022: in-hospital mortality rate, proportion of emergency procedures, average length of stay, mean cost per hospitalization, and population-adjusted surgical rate (procedures per 100,000 inhabitants).

**Table 2:** Summary of cardiovascular surgical indicators and syphilis incidence by Brazilian region between 2008 and 2022. Average CHS rates (population-adjusted), in-hospital mortality, costs, length of stay, and syphilis incidence across the five regions.

Region	Average syphilis incidence rate	Average CHS rate^1^	Average mortality rate of CHS	Average cost per CHS	Average length of stay per CHS
Central-West	4.17	35.8	7.6	3,598	12.6
Northeast	6.96	48.8	7.6	2,343	10.6
North	5.28	66.2	10.1	2,749	12.6
Southeast	7.96	44.3	6.6	3,664	11.2
South	6.67	59.2	7.4	3,447	9.7

These indicators reveal marked regional differences. For example, the North region exhibited the highest proportion of emergency procedures (66.2%) and the longest average hospital stay (10.1 days), suggesting both late presentation and limited access to elective surgical services. Conversely, the Southeast had the highest mortality rate (7.96%) despite a relatively shorter length of stay (6.6 days) and one of the highest average costs per hospitalization, pointing toward a complex interplay between case severity, resource allocation, and systemic efficiency.

The Northeast region, with the lowest cost per hospitalization (R$ 2,343) and a high emergency procedure rate (48.8%), continues to show signs of underfunding and reactive care models. Meanwhile, the South and Midwest regions presented intermediate profiles, with the South recording the lowest rate of procedures per 100,000 inhabitants (9.7), suggesting possible underutilization or supply–demand mismatch. Taken together, these data reinforce the need for regionally tailored health policies to address local disparities in surgical capacity, emergency responsiveness, and investment in elective cardiovascular care.

**Table 3:** Correlation matrix of the variables in Brazil between 2007 and 2023. Each cell represents the strength and direction of the linear relationship between two variables, with values ranging from −1 (perfect negative correlation) to +1 (perfect positive correlation). This matrix highlights significant associations, such as the moderate negative correlation between Syphilis Incidence Rate and Mortality Rate, and the strong negative correlation between Cost per Surgery and Mortality Rate.

**Variable**	**Syphilis incidence**	**Cost per surgery**	**Length of stay**	**Mortality rate**
**Syphilis incidence**	1.00	0.61	0.17	−0.48
**Cost per surgery**	0.61	1.00	0.38	−0.49
**Length of stay**	0.17	0.38	1.00	0.18
**Mortality rate**	−0.48	−0.49	0.18	1.00

**Table 4:** Ordinary least squares regression of mortality rate of cardiovascular congenital surgery. For each predictor, the table provides the regression coefficient (ß), standard error, t-statistic, *p*-value, and 95% confidence interval. The model explains 44.2% of the variance in mortality rates (*R*^2^ = 0.442), with significant predictors including Syphilis Incidence Rate (negative association) and Cost per Surgery (negative association).

**Variable**	Coefficient (ß)	Standard Error	T-statistic	*p*-value	95% Confidence Interval
**Constant**	6.680	1.010	6.614	<0.001	(4.67–8.69)
**Syphilis** ** incidence rate**	−0.135	0.058	−2.311	0.023	(−0.25 –−0.019)
**Cost per** ** surgery**	−0.001	0.000	−4.446	<0.001	(−0.001 –−0.001)
**Length of stay**	0.440	0.095	4.631	<0.001	(0.251–0.63)

To quantify the relationship between syphilis incidence and surgical outcomes, correlation analysis (Table 3) was conducted, revealing significant associations between key variables. The incidence rate of syphilis exhibited a moderate positive correlation with cost per surgery (*r* = 0.61), suggesting that regions with a higher syphilis incidence tend to incur higher treatment costs for congenital heart surgeries. This association may stem from the increased complexity of cases in high-incidence regions, which require more advanced interventions, longer hospital stays, and greater resource utilization. Conversely, the incidence rate of syphilis showed a moderate negative correlation with the mortality rate (*r* = −0.48), indicating that regions with higher syphilis incidence tended to have lower mortality rates. This counterintuitive finding could reflect an improved healthcare infrastructure and resource allocation in response to the growing burden of CS. Cost per surgery demonstrated a strong negative correlation with mortality rate (*r* = −0.49), underscoring the critical role of financial resources in reducing mortality. Higher treatment costs are likely to correspond to advanced medical technologies, skilled personnel, and better postoperative care, all of which contribute to improved outcomes. LOS had a weak positive correlation with mortality rate (*r* = 0.18), implying that prolonged hospital stays might be linked to poorer outcomes, potentially due to complications or adverse events during recovery.

Regression analysis (Table 4) further quantified these relationships using Ordinary Least Squares (OLS) regression, explaining 44.2% of the variance in mortality rates (*R*^2^ = 0.442). The constant term (*β* = 6.680, *p* < 0.001) represents the baseline mortality rate when all the predictors are zero. The incidence rate of syphilis was significantly negative (*β* = −0.135, *p* = 0.023), indicating that a one-unit increase in syphilis incidence was associated with a 0.135 percentage point decrease in mortality. This finding aligns with the correlation analysis and suggests that regions with a higher syphilis incidence may have implemented measures to improve surgical outcomes. Cost per surgery also had a significant negative coefficient (*β* = −0.001, *p* < 0.001), reinforcing the importance of resource allocation in reducing mortality. Every additional unit of cost per surgery was associated with a 0.001 percentage point reduction in mortality rate. LOS had a positive coefficient (*β* = 0.440, *p* < 0.001), confirming that longer hospital stays were associated with higher mortality rates. Residual analysis (Table 4) confirmed the validity of the OLS model, with the Shapiro–Wilk test (*p* = 0.280) indicating no significant deviation from normality, while the Durbin-Watson statistic (2.382) suggested no autocorrelation in residuals. The Jarque–Bera test (*p* = 0.162) further supported the normality assumption. However, the slight skewness (0.250) and kurtosis (3.883) indicated minor deviations from normality, which were not severe enough to invalidate the model.

Machine learning models (Table 6) complemented the regression analysis by exploring the nonlinear relationships and feature importance.

The Random Forest model achieved an *R*^2^ score of 0.321, capturing nonlinear interactions but explaining less variance than the OLS model. In contrast, the Linear Regression model outperformed the Random Forest model, achieving an *R*^2^ score of 0.513, confirming the dominance of linear relationships. The higher *R*^2^ observed in the simple linear regression reflects a strong individual association between the selected independent variable and the outcome. By contrast, the lower *R*^2^ in the multiple OLS regression is due to the inclusion of several explanatory variables, where multicollinearity and variance dilution may reduce the overall explanatory power. This difference highlights the tradeoff between model simplicity and comprehensiveness. Feature importance inferred from the regression coefficients highlights the critical roles of syphilis incidence rate (negative coefficient = −0.135), cost per surgery (negative coefficient = −0.001), and LOS (positive coefficient = 0.440) in predicting mortality rates. These findings suggest that while nonlinear interactions exist, they are less dominant than linear relationships. Together, these analyses provide a comprehensive understanding of the factors influencing congenital heart surgery outcomes, emphasizing the need for targeted interventions to address regional disparities and optimize resource allocation.

## Discussion

The findings of this study highlight the complex interplay between CS incidence and congenital heart surgery outcomes in Brazil, providing critical insights into regional disparities, healthcare resource allocation, and systemic challenges in neonatal cardiovascular care. By integrating population-level data from 2007 to 2023, this ecological analysis provides a nuanced understanding of how rising syphilis rates intersect with surgical mortality, costs, and LOS while highlighting broader implications for public health policy and clinical practice.

A striking finding was the negative correlation between CS incidence and surgical mortality rates (*r* = −0.48), corroborated by the OLS regression model (*β* = −0.135, *p* = 0.023). While it is associated with multisystem complications that could theoretically exacerbate surgical risks^18^, three mechanisms may explain this paradox. Regions with higher syphilis incidence, such as the Northeast and Central-West, demonstrated substantial surgical capacity growth (Figure 2), likely driven by investments in neonatal care infrastructure to address rising congenital heart defect (CHD) burdens^19^. For instance, the surgical volume of the Northeast nearly doubled (2007–2023), coinciding with a decline in mortality from 9.2% to 6.8%. This aligns with evidence that concentrated resource allocation in high-burden regions can mitigate adverse outcomes despite rising disease incidence^20^. Heightened syphilis screening in high-incidence regions may enable earlier maternal treatment with benzathine penicillin, reducing the severity of cardiac anomalies that require surgery^21^. Studies have confirmed that early prenatal intervention significantly lowers the risk of severe CS complications, including structural heart defects^22^. Aggregated regional data may mask individual-level risks. Infants with syphilis-related cardiac defects could still face higher mortality, but this effect may be diluted in regions with robust surgical programs^23^.

This negative association contrasts with cohort studies in high-syphilis settings, such as South Africa, where CS-positive infants had a 2.3-fold higher postoperative mortality risk^24^. This discrepancy underscores the role of Brazil’s unified healthcare system (SUS) in buffering syphilis impacts through equitable resource distribution^25^. However, ecological design limits causal inferences, necessitating individual-level studies to disentangle direct syphilis effects from regional healthcare dynamics^26^.

The strong negative correlation between cost per surgery and mortality (*r* = −0.49, *β* = −0.001, *p* <0.001) underscores the critical role of financial investment in surgical outcomes. Higher costs likely reflect access to advanced technologies (e.g., ECMO, hybrid operating rooms), which reduce mortality in complex CHD cases^27^. For example, Southeast Region, with the highest average cost per surgery (R3,534), achieved a mortality rate of 6.53,534). This aligns with global data linking higher per capita healthcare expenditure to lower postoperative mortality in LMICs^28^. However, diminishing returns were evident in Central-West, where a 16-fold syphilis increase (Figure 1) yielded only a modest mortality reduction (8.1% to 7.4%), likely due to systemic inefficiencies (e.g., delayed referrals and staff shortages)^29^.

Interestingly, the analysis revealed a negative correlation between syphilis incidence and hospital mortality rates. This unexpected relationship may be explained by differences in regional health system performance. Areas with stronger primary healthcare infrastructure and more comprehensive disease surveillance systems are more likely to detect and report cases of syphilis, thus increasing the incidence rate in administrative data. These same regions may also offer better inpatient care, contributing to reduced mortality. Thus, syphilis incidence may act as a proxy for public health service coverage.

Regions with a higher CS incidence often implement intensified prenatal screening programs, which inadvertently improve the early detection of CHDs. For example, Brazil’s Family Health Strategy, a community-based primary care program, has increased access to fetal ultrasound and maternal syphilis testing in high-burden areas. Early diagnosis allows for timely maternal penicillin treatment and preoperative stabilization of infants, thereby reducing surgical risks. A study by Leal et al. found that states with >80% prenatal care coverage had 30% lower odds of severe CHD complications at birth, even amid rising syphilis rates^30^.

Investments in primary care infrastructure in CS hotspots, such as Brazil’s Northeast, have strengthened maternal-child health networks. Macinko and Harris demonstrated that regions with robust Family Health Strategy coverage saw a 24% reduction in neonatal mortality from congenital anomalies, as integrated care teams coordinate syphilis management and cardiac surgery referrals. This “horizontal integration” buffers surgical mortality despite high CS incidence^31^.

Conditional cash transfer programs (e.g., Brazil’s *Bolsa Família*) indirectly improve surgical outcomes by addressing socioeconomic determinants. Rasella et al. linked these programs to a 17% decline in infant mortality from congenital anomalies in low-income municipalities. By reducing malnutrition and improving healthcare access, such policies mitigate comorbidities (e.g., sepsis, prematurity) that elevate surgical risks in syphilis-affected infants^32^.

Regions with high CS incidence often centralize congenital heart surgeries to optimize resource use. Welke et al. showed that Brazilian hospitals performing >150 annual pediatric cardiac surgeries achieved 22% lower mortality than low-volume centers^33^. Centralization in states such as Pernambuco, a CS hotspot, has concentrated on expertise, advanced technologies (e.g., hybrid operating rooms), and standardized protocols, offsetting individual risks, phenomena that could have occurred to reduce the flow of patients from the central-west region^33^.

Regions with rigorous CS reporting systems often have better mortality data capture, thereby reducing underestimation of survivable cases. Silveira et al. (2021) noted that São Paulo’s real-time syphilis surveillance correlates with 95% completeness in CHD outcome reporting, while under-resourced regions miss 40% of postoperative deaths. This surveillance bias creates an inverse correlation between CS incidence and mortality^34^.

Machine learning models highlighted nonlinear interactions, identifying a “cost ceiling” beyond which additional expenditures yielded minimal mortality reductions. Similar patterns in LMICs suggest that infrastructure gaps limit financial efficacy without parallel workforce training and supply chain improvements^35^. Holistic interventions, such as the World Heart Federation’s surgical training programs, are critical for sustainable outcomes^36^.

The positive association between LOS and mortality (*β* = 0.440, *p* <0.001) suggests prolonged hospitalizations signal complications rather than improved care. Infants with postoperative infections or multi-organ dysfunction often require extended stays, which independently increases mortality risk^37^. A Brazilian multicenter study found that LOS >14 days increased mortality by 40%, primarily due to nosocomial infections^38^. Regional discharge practices further confound this relationship: urban centers like Southeast may prioritize early discharges due to bed shortages, whereas rural regions lack step-down facilities, prolonging LOS^39^.

CS exacerbates LOS through comorbid conditions (e.g., hepatitis, osteitis), delayed recovery, and increased infection susceptibility^40^. A Peruvian study reported a median LOS of 21 days for syphilis-positive infants versus 12 days for non-syphilitic infants, with sepsis explaining 60% of the disparity^41^. Integrated care pathways addressing cardiac and infectious comorbidities preoperatively could reduce LOS and mortality^42^.

Stark regional disparities reflect broader inequities in Brazil’s healthcare system. While Northeast and Southeast achieved mortality rates <7%, North Region’s rate remained elevated at 9.8%, driven by geographic isolation, socioeconomic deprivation, and limited prenatal screening^43^. Only 62% of pregnant women in Nort receive adequate syphilis testing versus 89% in Southeast, perpetuating cycles of undiagnosed infections and advanced CHDs^44^. This aligns with the “inverse care law,” where populations with the greatest needs receive the poorest care^45^.

Paradoxically, high-syphilis regions like Northeast demonstrated robust surgical growth (Figure 2), reflecting policy-driven resource reallocation. Brazil’s 2010 National Policy for Neonatal Cardiac Surgery prioritized funding for high-CHD-prevalence regions, including syphilis hotspots^46^. However, Central-West’s stagnant mortality rates along with surgical decrease highlight the insufficiency of funding alone without addressing upstream determinants (maternal education and prenatal access)^47^.

The apparent decrease in congenital heart surgeries in the Central-West Region deserves closer scrutiny, especially considering statements suggesting expanded surgical capacity. This trend may reflect not a true contraction in services, but rather changes in referral flows, temporary funding cycles, or the centralization of surgeries in a few specialized centers not uniformly distributed across the region. It may also suggest that early efforts to expand capacity in regions with higher syphilis incidence, such as the Northeast and Central-West, were not sustained over time. When viewed alongside the stagnation in the North and South, these findings reinforce concerns about persistent inequities in the allocation of resources and the continuity of care. Ultimately, they highlight the need for long-term investment strategies that not only expand infrastructure, but also ensure equitable and sustained access to congenital heart surgery across all regions.

Ecological design precludes causal inferences and individual-level risk stratification^48^. Underreporting syphilis cases in rural regions may bias associations^49^, whereas administrative data inaccuracies (e.g., misclassified surgical outcomes in SIH-SUS) introduce measurement errors^50^. Focusing on mortality, costs, and LOS overlooks critical outcomes, such as neurodevelopmental sequelae, which are increasingly recognized in CHD research^51^.

**Table 5:** Residual analysis of the regression model. This table summarizes diagnostic tests conducted to evaluate the validity and assumptions of the Ordinary Least Squares (OLS) regression model. Key tests include the Shapiro–Wilk Test for normality of residuals (*p* = 0.280), the Durbin-Watson Statistic for autocorrelation (value = 2.382), and the Jarque–Bera Test for normality (*p* = 0.162). Additional metrics such as skewness (0.250) and kurtosis (3.883) are also reported. These diagnostics confirm that the model meets the assumptions of linear regression, with no significant deviations from normality or autocorrelation.

**Test/Statistic**	**Value**	***p*-value**
**Shapiro–Wilk Test**	0.982	0.280
**Durbin-Watson Statistic**	2.382	
**Jarque–Bera Test**	3.644	0.162
**Skewness**	0.250	
**Kurtosis**	3.883	

**Table 6:** Machine Learning model performance. This table compares the performance of two machine learning models—Random Forest and Linear Regression—in predicting mortality rates of congenital heart surgeries. These results indicate that linear relationships dominate the data, as the simpler Linear Regression model explained more variance in mortality rates than the Random Forest model.

**Model**	Mean squared error	*R*^2^ score
**Random Forest**	4.64	0.321
**Linear Regression**	3.33	0.513

Residual diagnostics further support the robustness of the regression model, despite minor deviations from ideal conditions. As shown in Table 5, the residuals displayed acceptable normality (Shapiro–Wilk = 0.982, *p* = 0.280; Jarque–Bera = 3.644, *p* = 0.162), no autocorrelation (Durbin-Watson = 2.382), and minimal skewness (0.250), reinforcing the validity of the linear regression assumptions. However, the kurtosis value of 3.883, which is slightly above the mesokurtic benchmark of 3, suggests a modest leptokurtic distribution. This indicates a slightly higher concentration of values near the mean and in the tails, which could reflect the presence of rare but influential regional deviations in mortality outcomes. In the context of population-level health data, such kurtosis may be attributable to structural outliers, regions with either particularly efficient surgical systems or disproportionately limited access. Importantly, these findings do not undermine the model’s integrity, but instead highlight the heterogeneous nature of Brazil’s healthcare landscape, reinforcing the need for regional stratification in future analyses.

CS resurgence demands dual focus on prevention and surgical preparedness. Strengthening prenatal screening in high-incidence regions could reduce syphilis-related CHDs and the surgical burden^52^. Brazil’s 2016 syphilis elimination plan, which expanded rapid testing and partner notification, offers an LMIC model, although decentralized strategies are needed for equitable implementation^53^.

Surgically, tiered care networks centralizing complex CHD cases in high-volume centers, while decentralizing prenatal/postnatal care, could optimize outcomes. Mexico’s regionalized referral systems have reduced mortality by 22% through telemedicine-supported follow-up^54^. Cost-effectiveness analyses should prioritize high-impact interventions such as preoperative nutritional support for syphilis-positive infants^55^.

While ecological studies provide valuable insights into population-level trends, their utility in drawing individual-level inferences is inherently limited. Ecological fallacy, a core concern, arises when group-level associations are erroneously assumed to apply to individuals, potentially masking true causal relationships. For example, a region with a high CS incidence and low surgical mortality may reflect systemic factors (e.g., better healthcare infrastructure) rather than a protective effect of CS on individual infants. Additionally, unmeasured confounding factors, such as regional variations in prenatal care quality or unrecorded comorbidities, and surveillance biases, such as differential reporting of CS and surgical outcomes across regions, can distort observed associations. Ecological designs also lack temporal precision, as they often analyze exposures and outcomes concurrently, rather than accounting for biologically relevant lags (e.g., maternal infection timing relative to surgery). These limitations underscore the need for caution when extrapolating findings to individual risks and highlight the importance of corroborating ecological results with patient-level cohort or linkage studies.

To build on the ecological associations identified in this study, future research should incorporate individual-level data to better understand the complex interplay between CS and congenital heart surgery outcomes. Prospective cohort studies tracking maternal syphilis status, infant cardiac anatomy, surgical details, and postoperative complications would help disentangle the direct effects of CS on regional healthcare disparities. Additionally, multicenter collaborations could standardize data collection on critical confounders, such as prematurity, birth weight, and nutritional status, factors that likely contribute to unexplained outcome variance.

Further studies should explore the role of healthcare system factors in mitigating CS-related surgical risks. Comparative analyses of high-performing versus under-resourced regions could identify the best practices in neonatal cardiac care, including optimal staffing ratios, surgical volume thresholds, and postoperative ICU protocols. Qualitative research examining referral pathways and delays in high-CS burden areas may reveal modifiable barriers to timely interventions. The paradoxical association between the incidence of CS and reduced mortality warrants further investigation. Individual-level risk stratification can clarify whether this finding reflects true system-level resilience or surveillance bias. Advanced modeling techniques such as machine learning with causal inference methods may help quantify the relative contributions of prenatal screening, surgical centralization, and socioeconomic factors. Such evidence would inform targeted policies to improve outcomes in regions where the CS and CHD burdens intersect.

## Conclusions

This study investigated the influence of congenital syphilis incidence on congenital heart surgery outcomes in Brazil, focusing on mortality rates, treatment costs, and length of hospital stay. Using an ecological study design and publicly available health data from 2007 to 2023, the research highlights alarming increases in syphilis incidence across all regions, with significant regional disparities in healthcare resource allocation and surgical outcomes.

Key findings revealed a nearly 16-fold increase in syphilis incidence in the Central-West Region, while Northeast and North regions also experienced substantial growth. These trends correlate with variations in surgical capacity, as regions like the Northeast and Southeast expanded their number of approved surgeries, unlike the North and South, where growth was slower. Correlation analysis identified significant relationships: syphilis incidence showed a moderate negative correlation with mortality rate (*r* = −0.48) and a positive correlation with cost per AIH (*r* = 0.61), highlighting the financial and operational strain in healthcare systems.

Regression modeling explained 44.2% of the variance in mortality rates, with syphilis incidence (*β* = −0.135) and cost per AIH (*β* = −0.001) showing significant negative associations with mortality, while length of stay (*β* = 0.440) positively correlated with poorer outcomes. Machine learning models confirmed the dominance of linear relationships, with Random Forest achieving an *R*^2^ score of 0.321 and Linear Regression outperforming it by 0.513.

These results underscore the need for targeted interventions to address regional inequities and optimize resource allocation. While this study cannot establish causality, it provides actionable insights for policymakers and healthcare providers to improve neonatal health outcomes. By adhering to ethical guidelines and methodological rigor, this research lays the groundwork for future studies and public health strategies aimed at reducing the burden of congenital syphilis and enhancing surgical outcomes in Brazil.
